# To: Ventriculitis incidence and outcomes in patients with aneurysmal subarachnoid hemorrhage: a prospective observational study

**DOI:** 10.62675/2965-2774.20250083

**Published:** 2025-10-22

**Authors:** Josef Finsterer, Carla Alessandra Scorza, Fulvio Alexandre Scorza

**Affiliations:** 1 Neurology and Neurophysiology Center Department of Neurology Vienna Austria Department of Neurology, Neurology and Neurophysiology Center - Vienna, Austria.; 2 Universidade Federal de São Paulo São Paulo SP Brazil Universidade Federal de São Paulo - São Paulo (SP), Brazil.

## To the Editor

We were interested to read the article by Turon et al. on a prospective study of 271 patients who were admitted to the intensive care unit (ICU) between July 2015 and December 2020 for aneurysmal subarachnoid hemorrhage (SAH) and required external ventricular drainage (EVD).^( 1 )^ Of the patients included in the study, 127 developed an EVD-associated infection with a median latency between SAH and EVD of 8.8 days and a median latency between EVD and infection of 4.4 days.^( 1 )^ The modified Rankin Scale (mRS) and mortality did not differ between patients with and without infection at discharge and 12-month follow-up.^( 1 )^ The study concluded that EVD-associated infection occurs in half of SAH patients receiving EVD, but there is no difference in outcome between patients with and without EVD-associated infection.^( 1 )^ The study is impressive, but some points should be discussed.

The first point is that the risk factors for EVD and consecutive infections have not been specified.^( 1 )^ Therefore, we should know whether age, gender, comorbidities, concomitant medications, severity of bleeding, blood volume, ventricular intrusion, or intraventricular thrombolysis influenced the indication for EVD and infection rate as well as the outcome.

The second point is that the study covered the beginning of the severe acute respiratory syndrome coronavirus 2 (SARS-CoV-2) infection (SC2I) from by July 2015 to December 2020.^( 1 )^ Therefore, we should know how many patients enrolled during this period also had SC2I and whether the spectrum of infectious agents and outcome differed between the patients with and without SC2I.

The third point is that the occurrence of ischemic stroke as a complication of SC2I was assessed clinically but not by imaging in every patient.^( 1 )^ It is not clear how it could be determined whether the occurrence of focal neurological deficits or deterioration of consciousness was due to ischemic stroke, relapse of SAH, infection, or intracerebral hemorrhage.

The fourth point is that the latency period between admission and treatment of the aneurysm (coiling, clipping, or no treatment) has not been determined.^( 1 )^ Need for EVD may depend not only on the type of aneurysm treatment, but also on the latency between SAH and therapy.^( 2 )^ Data should be re-analyzed to determine the influence of treatment modality and latency between SAH and therapy on infection rate and outcome. It would also be interesting to know whether the infection rate and outcome differed between patients with or without ventricular intrusion and those with or without intraventricular thrombolysis.

The fifth point is that it was not stated how many of the patients with SAH were diagnosed by the detection of xanthochromia on cerebrospinal fluid examination and in whom the cerebral computed tomography was negative.^( 3 )^ Were these patients actually diagnosed with an aneurysm, and if so, how, and did these patients have a definite onset of disease > 30 days prior to admission?

The sixth point is the discrepancy between the first sentence of the methods section, which states that all consecutive patients were included, and the statement that patients in whom SAH occurred > 30 days before admission, pregnant women, and those with a life expectancy < 48 hours were excluded. This discrepancy should be corrected. How could it be determined that patients would die within 48 hours? Why were pregnant women excluded?

The seventh point is that telephone interviews, not clinical visits, assessed the 12-month outcome. Telephone interviews have the disadvantage that the accuracy of the information cannot be easily verified, that it is not sure that the patient himself is answering the questionnaire and not a relative or caregiver, and that the calculated mRS is actually correct.^( 4 )^

In summary, this interesting study has limitations that put the results and their interpretation into perspective. Addressing these limitations could strengthen the conclusions and corroborate the study's message. Since the outcome of EVD-associated ventriculitis after aneurysmal SAH depends on many factors, it can only be adequately assessed if these are included in the analysis.

## Availability of data and material

All data are available from the corresponding author
